# Development of microsatellite markers for identifying Brazilian *Coffea arabica* varieties

**DOI:** 10.1590/S1415-47572010005000055

**Published:** 2010-09-01

**Authors:** Elisa S. N. Vieira, Édila V. de R. Von Pinho, Maria G. G. Carvalho, Danny G. Esselink, Ben Vosman

**Affiliations:** 1 Cooperativa Central de Pesquisa Agrícola, Núcleo de Biotecnologia, Cascavel, PRBrazil; 2 Universidade Federal de Lavras, Departamento de Agricultura, Lavras, MGBrazil; 3 Plant Research International, Wageningen UR Plant Breeding, WageningenThe Netherlands

**Keywords:** SSR, coffee, genetic similarity, molecular marker

## Abstract

Microsatellite markers, also known as SSRs (Simple Sequence Repeats), have proved to be excellent tools for identifying variety and determining genetic relationships. A set of 127 SSR markers was used to analyze genetic similarity in twenty five *Coffea arabica* varieties. These were composed of nineteen commercially important Brazilians and six interspecific hybrids of *Coffea arabica*, *Coffea canephora* and *Coffealiberica.* The set used comprised 52 newly developed SSR markers derived from microsatellite enriched libraries, 56 designed on the basis of coffee SSR sequences available from public databases, 6 already published, and 13 universal chloroplast microsatellite markers. Only 22 were polymorphic, these detecting 2-7 alleles per marker, an average of 2.5. Based on the banding patterns generated by polymorphic SSR loci, the set of twenty-five coffee varieties were clustered into two main groups, one composed of only Brazilian varieties, and the other of interspecific hybrids, with a few Brazilians. Color mutants could not be separated. Clustering was in accordance with material genealogy thereby revealing high similarity.

## Introduction

Coffee is an important crop in several countries. Of all the species, *Coffea arabica* L. is the most widely grown, due both to the low caffeine content and the smooth final beverage. This species accounts for almost the entire production of Latin American countries (Orozco-Castillo *et al.*, 1994).

Traditionally, morphological and biochemical characteristics have been used to characterize varieties. Although these markers are still important, they are somewhat limited, through the need for physical space for evaluation, the effect of environmental conditions on character expression, and the time required for making a full description, as several characters need to be evaluated during the entire growth period of the plant. For coffee trees, the latter limitation is extremely relevant, through being a perennial crop requiring three-years-growth until full maturity (Mendes and Guimarães, 1998). It takes at least fifteen years to obtain a new variety.

In 2001, *C. arabica* was included on the Brazilian roll of species from which varieties can be protected, without, however, indicating stable and homogeneous markers required for the effective enforcement of protective measures. In the past, DNA-based markers have been used for studying genetic diversity in many plant species. This type of marker, besides facilitating the analysis of variation present in DNA itself, can also be used for variety identification. In addition, they are environmentally independent, and may be detected in any type of tissue and developmental phase of the plant (Arens *et al.*, 1995; Ferreira and Grattapaglia, 1998). Analysis of *C. arabica* varieties in Brazil has revealed that the material employed is derived from few ancestral varieties (Typica, Bourbon and Sumatra), which themselves have undergone mutual spontaneous mutations and crossings (Mendes and Guimarães, 1998).

Nuclear DNA variation in coffee has been evaluated by using molecular markers such as RFLP (Lashermes *et al.*, 1999), RAPD (Diniz *et al.*, 2005; Anthony *et al.*, 2002, Silveira *et al.*., 2003), AFLP (Steiger *et al.*, 2002; Anthony *et al.*, 2002) and SSRs (Combes *et al.*, 2000; Anthony *et al.*, 2002; Moncada and McCouth 2004; Maluf *et al.*, 2005; Poncet *et al.*, 2006; Aggarwal *et al.*, 2007; Silvestrini *et al.*, 2007), whereby it has been shown that genetic variation in the genus *Coffea* is low, especially among cultivated *C. arabica* tetraploid varieties. Chloroplast DNA (cpDNA) non-coding regions have been used as a source of molecular markers in studies concerning the relationships within and among species of this genus (Orozco-Castillo *et al.*, 1996; Cros *et al.*, 1998), where only interspecific polymorphism was detected.

Simple sequence repeats (SSR), or microsatellite markers, are very attractive for studies in plant genetics, through their usefulness in evaluating those varieties with a narrow genetic base (Bredemeijer *et al.*, 2002). Furthermore, they can be efficiently analyzed by rapid and simple polymerase chain reactions, besides being co-dominant, highly reproducible and multi-allelic, and capable of being automated (Ferreira and Grattapaglia, 1998).

By using markers developed for *C. arabica*, Moncada and McCouth (2004) showed the particular value of SSR markers for discriminating closely related commercial varieties of coffee. Maluf *et al.* (2005) and Silvestrini *et al.* (2007) confirmed the low genetic diversity in coffee, mainly in tetraploid varieties, although none were related to the varieties under study.

The number of microsatellite markers currently available for coffee remains limited. To date, only 224 genomic SSR markers for species of the *Coffea* genus have been described (Hendre *et al.*, 2008). *Coffea arabica* is the most important, and there is an urgent need for additional microsatellite makers for facilitating the identification of closely related varieties. Thus, the aim was to develop and characterize additional microsatellite markers for *C. arabica*, and evaluate their use in identifying varieties of commercial interest in Brazil.

## Material and Methods

###  Plant material and DNA isolation

A set of 19 *Coffea arabica* varieties was selected, these representing all the major varieties grown in Brazil (Table 1). The DNA of each genotype was extracted from ground seeds. Six interspecific hybrids of *C. arabica*, *C. liberica* and *C. canephora* from the Centro de Investigação das Ferrugens do Cafeeiro (CIFC) were included in this work. The DNA of this material was extracted from freeze-dried leaves. For the construction of genomic libraries enriched for microsatellites, DNA was extracted from leaves of variety Catuaí Vermelho IAC-44 (*C.arabica*). In all cases the DNA extraction was carried out using the DNeasy Plant Mini Kit (Qiagen), according to manufacturer's instructions.

###  SSR sequences from public databases and primer design

Coffee microsatellite sequences were extracted from the NCBI database. Those containing di-nucleotide (n > 10) or tri-nucleotide (n > 6) repeats were selected for primer design. PCR primers flanking the repeat sequence were designed using the primer select module of the DNAstar Lasergene package. Six SSRs for *C. arabica* described by Combes *et al.* (2000) and 13 cpDNA SSRs (Taberlet *et al.*, 1991; Orozco-Castillo *et al.*, 1996) were also tested.

###  Microsatellite isolation

Additional microsatellites were isolated from enriched small-insert genomic libraries constructed according to Van de Wiel *et al.* (1999), with a minor modification. DNA of the Catuaí Vermelho IAC-44 variety was digested with *Alu*I, *Rsa*I, *Mbo*I or *Taq*I enzymes instead of being sonicated. After digestion, the DNA fragments were hybridized to filters containing the following synthetic oligonucleotides: (TCT)_10_, (TGT)_9_, (GAG)_8_, (GTG)_8_, (TGA)_9_, (AGT)_10_, (CGT)_8_, (GCT) _8_, (CT)_12_ and (GT)_12_. Filters, on being washed with 0.5xSSC 1% SDS (low stringency wash) and 0.2xSSC 1% SDS (high stringency wash), gave rise to two genomic libraries.

###  Nomenclature

The newly developed markers were named according to the nomenclature proposed by Hendre *et al.* (2008) for *C.canephora* SSR markers. Each marker was identified by the suffix CarM, indicating *C. arabica* microsatellite marker, followed by a number.

###  Microsatellite analysis

Microsatellites were amplified by PCR in a 20 μL reaction volume, containing 10 mM of Tris-HCl pH 9.0, 20 mM of (NH_4_)_2_SO_4_, 0.01% Tween 20, 1.5 mM MgCl_2_, 0.1 mM of each dNTP, 4 pmol of each primer, 0.2 units of Goldstar Taq DNA polymerase (Eurogentec, Maastricht, The Netherlands), and 16 ng of genomic DNA. The amplifications were performed in a PTC-200 MJ Research Thermal Cycler, programmed for one step at 94 °C for 3 min, followed by 30 cycles (30 s at 94 °C, 30 s at the annealing temperature determined for each primer pair, and 45 s at 72 °C), and a final extension at 72 °C for 3 min. All primers were synthesized by Eurogentec (Maastricht, The Netherlands) (Table S1).

The PCR products were separated on 6% polyacrylamide gels, by using a Sequi-Gen Sequencing Cell (Bio-Rad) apparatus at 110W for 1-3 h in 1x TBE buffer. After electrophoresis, the products were visualized through silver staining as described by Van de Wiel *et al.* (1999), and the patterns analyzed for the presence of polymorphism and the quality of the banding pattern, according to Arens *et al.* (1995).

###  Data analysis

For polymorphic microsatellite loci, the number of alleles per locus and allelic phenotypes were counted. Considering that *C. arabica* is a tetraploid species, assessing the actual genotype itself based on band intensity is unreliable. Therefore, banding patterns were observed for each polymorphic locus and recorded as allelic phenotypes (Becher *et al.*, 2000). In order to quantify the discrimination power of the microsatellite markers, the number of effective alleles (ne) for each marker was calculated according to the formula (Hartl and Clark, 1997): ne = 1/Σ (E/F)^2^, where E is the total number of genotypes with each allele of locus *i*, and F is the total number of alleles of the locus *i* in all genotypes.

A presence/absence (1/0) allele matrix was built, and Jaccard similarity was calculated by using the NTSYS (version 2.1) computer program. UPGMA dendrogram was calculated using the SHAN algorithm of the NTSYS package. Bootstrapping was applied to evaluate the degree of association between the genetic similarity matrix and dendrogram, using the BOOD software version 3.0 (Coelho, 2001). Pearson correlationship was calculated using the GENES software Windows version (Cruz, 2001) to indicate the extent to which the clustering of genotypes demonstrated in the dendogram accurately represents the estimates of genetic similarity.

## Results

###  Microsatellite enrichment from *C. arabica*

An overview of the results obtained with microsatellite enrichment procedures is given in Table 2. An arbitrary number of positive clones were sequenced. Of the 135 recombinant clones obtained from the first enrichment (low stringency library), 110 were sequenced, with 41% (45) containing a microsatellite sequence, 2 of which redundant. Twenty microsatellite sequences had perfect repeats, 22 had imperfect repeats and three were compound repeats. Flanking regions in eighteen inserts were large enough for primer design. From the 397 recombinant clones characterized in the second enrichment (high stringency library), 192 were sequenced, with 46% (89) containing microsatellite sequences, 14 of which redundant. For this set, 51 microsatellite sequences were perfect repeats and 29 imperfect and nine were compound repeats. Flanking regions in 35 inserts were suitable for primer design.

In total, 53 primer pairs could be developed, 23 for di-, 24 for tri-, 3 for tetra-, 2 for penta- and 1 for compound-nucleotide repeats. The latter two were found only in the first enriched library. The sequences of all markers obtained are shown in Table S1. Compound microsatellite sequences consisting of CCA/TCA and TGA/GAA repeats were found in both libraries, whereas clones containing microsatellite sequences which were not specifically searched for, as GGA, were found in the first. GT and TGA were the most common di- and tri-nucleotide motifs encountered (results not shown). All 53 primer pairs produced a clear PCR fragment.

###  Markers from public database sequences and literature

The screening of a public (NCBI) database for microsatellite sequences resulted in 56 accessions which met the set criteria (more than 6 repeat units per tri-nucleotide repeat and 10 repeat units per di-nucleotide). Some of these sequences had already been used for marker development. Nevertheless, as primer design was undertaken independently, there are differences in the primers used to amplify SSR markers in our study and theirs (Coulibaly *et al.*, 2003; Poncet *et al.*, 2006). An additional 6 primer pairs were available from literature (Combes *et al.*, 2000). These sequences are also incorporated in Table S1. Beside the nuclear DNA markers, 13 cpDNA primers were tested (Taberlet *et al.*, 1991; Orozco-Castillo *et al.*, 1996).

###  Marker characterization and allelic variation

**Figure 1:**
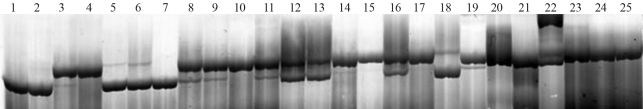
Molecular pattern obtained with the marker CarM092 (1: Acaiá Cerrado MG1474; 2:Mundo Novo IAC 376-4; 3:Obatã IAC 1669-20; 4: Oeiras MG6851; 5: Ouro Verde IAC H5010-5; 6: Rubi MG1192; 7: Topázio MG1190; 8: Bourbon Amarelo IAC J22; 9: Bourbon Vermelho IAC 662; 10: Catuaí Amarelo IAC 62; 11: Catuaí Vermelho IAC 99; 12:Catucaí Amarelo 2015/cova479; 13: Catucaí Vermelho 2015/cova476; 14: Caturra Amarelo IAC 476; 15: Caturra Vermelho IAC 477; 16: IAPAR 59; 17: Tupi IAC 1669-33; 18: Icatu Amarelo IAC2944; 19: Icatu Vermelho IAC 2945; 20: CIFC H147/1; 21: CIFC 34/13(S353-4/5); 22: CIFC 832/1; 23: CIFC1343/269; 24: CIFC 110/5; 25: CIFC H539/8.

A total of 127 primer pairs were tested for pattern quality and degree of polymorphism using a set of 19 coffee-varieties and 6 inter-specific hybrids. 125 primers amplified the expected DNA fragments, although only 22 were polymorphic. Most markers contained a GT repeat. All polymorphic markers gave a pattern quality of 1 or 2 (Arens *et al.*, 1995) and could be scored unambiguously. An example of the molecular pattern obtained with the CarM092 marker is shown in Figure 1. A total of 55 alleles were detected using the 22 polymorphic SSR loci, the number of alleles per locus ranging from 1 to 7, an average of 2.5 alleles per locus (Table 3).

###  Variety identification

**Figure 2:**
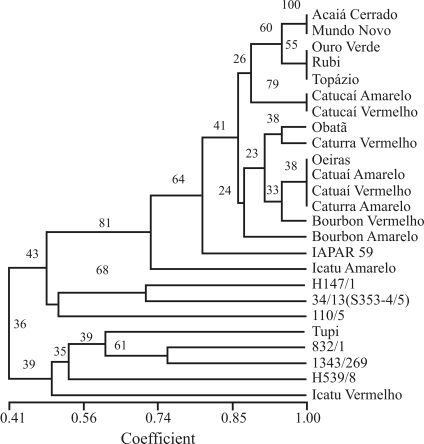
UPGMA dendrogram obtained using the Jaccard similarity of 19 coffee varieties and 6 interspecific hybrids, with data from 22 polymorphic microsatellite loci (Bootstrap values are in percentages).

The 22 polymorphic SSR markers were used to group the set of varieties and inter-specific hybrids. The UPGMA dendrogram revealed that most of the Brazilian varieties were placed in a group with high bootstrap value (81.7%), thereby indicating reliable clustering (Figure 2). The interspecific hybrids and two Brazilian varieties (Tupi and Icatu Vermelho) were placed in groups with bootstrap values below 50%. Pearson correlation was 0.958, thus indicating that the observed clustering of varieties in the dendrogram accurately represented the estimates of genetic similarity.

The allelic profiles of all the varieties used in this study can be seen in Table S2. As regards Brazilian material, variety-specific alleles were detected with the markers CarM101, CarM051 and CarM052 for the varieties Bourbon Vermelho, Icatu Amarelo and Vermelho.

The loci CarM068, CarM086, CarM002, ccmp3, ccmp10 and NTCP8 amplified only in interspecific hybrids.

The CarM051 locus was the most discriminating, with six allelic phenotypes and 3.4 effective alleles (Table 3). Although for CarM092 the effective alleles count was low, with only four allelic phenotypes, it was, together with CarM101, CarM051 and CarM052, one of the more discriminating markers for Brazilian varieties.

## Discussion

###  Enriched libraries

Enrichment based on the hybridization of genomic DNA fragments to filters containing synthetic oligonucleotide repeats, has been shown to be an efficient way of microsatellite retrieval in several species like tomato, lettuce, roses and *C*. *canephora* (Vosman and Arens, 1997; Van de Wiel *et al.*, 1999; Esselink *et al.*, 2003; Hendre *et al.*, 2008). Results presented in this paper indicated efficacy also for *C. arabica*.

In terms of efficiency, the second library (high stringency filter washing) generated a higher frequency of clones containing microsatellites, which, besides being longer, consisted of a higher percentage of perfect repeats. Most of the di-nucleotide microsatellite repeats were of the GT motif, which is in agreement with previous microsatellite retrieval efforts in coffee (Vascotto *et al.*, 1999), and with the *C. arabica* microsatellite sequences present in the NCBI database. In contrast, Ruas *et al.* (2003) showed that the GA-nucleotide motif, combined with other di, tri and tetra-nucleotide motifs, produced a high number of DNA fragments, thereby inferring a high frequency of poly GA microsatellite motifs in the coffee genome. It is well known that di-nucleotide repeats are very common in plants (Morgante and Olivieri, 1993). In *C. canephora*, the most common was di-nucleotide repeats (AT and AG) (Hendre *et al.*, 2008), which is in agreement with our results. The TGA motif was the most common among the tri-nucleotides, whereas in *C. canephora*, this was AGC (Hendre *et al.* 2008). Clones containing the TGA motif were also found by Vascotto *et al.* (1999) in coffee and other species, such as tomatoes and roses (Esselink *et al.*, 2003; He *et al.*, 2003). In *Arabidopsis thaliana* (Depeiges *et al.*, 1995), sugarcane (Cordeiro *et al.*, 2000) and black poplar (Van der Schoot *et al.*, 2000), the frequency of this motif was lower. In general SSR markers developed for *Coffea* sp. are mainly comprised of di and tri-nucleotide repeats (Poncet *et al.*, 2006; Aggarwal *et al.*, 2007).

###  Allele variation

In the set of coffee varieties and interspecific hybrids, only 22 (17%) out of the 127 markers tested were polymorphic, thereby clearly revealing the narrow genetic base of coffee. There was little diversity among the material tested, especially among the Brazilian varieties. The number of alleles per locus ranged from 1 to 7, which is in agreement with previous studies (Vascotto *et al.*, 1999; Anthony *et al.*, 2002; Moncada and McCouth 2004; Aggarwal *et al.*, 2007; Hendre *et al.*, 2008).

Moncada and McCouch (2004) used a set of 34 SSR markers to distinguish closely related commercial varieties of *C. arabica*, thereby confirming the need for working with SSR marker sets, in the case of crops with a narrow genetic base.

On considering the low level of polymorphism detected with isolated microsatellites, an attractive strategy could be to first try selecting SSRs with a high chance of being polymorphic. Recently, a software tool for identifying such SSRs in EST sequences was developed (Tang *et al.*, 2008). With more than 55,000 ESTs in the database (NCBI, December 2008), it was possible to identify several promising SSRs based on coding regions (Poncet *et al.*, 2006; Aggarwal *et al.*, 2007).

###  Variety identification

As already mentioned, *C. arabica* varieties are highly similar to each other. This high genetic similarity is possibly a consequence of the self-pollinating nature of *C. arabica*, as well as the breeding strategies used for coffee (Lashermes *et al.*, 1999; Combes *et al.*, 2000; Anthony *et al.*, 2002; Steiger *et al.*, 2002; Ruas *et al.*, 2003; Moncada and McConch 2004; Maluf *et al.*, 2005).

The interspecific hybrids clustered far from most of the Brazilian varieties probably because of the presence of *C. canephora* and *C. liberica* in the genealogy of these genotypes. This could be confirmed by using chloroplast markers, which detect only inter-specific variation (Table S2). The same was shown by Orozco-Castillo *et al.* (1996) in a study of taxonomic relationships within the genus *Coffea*, when using chloroplast DNA markers. Taberlet *et al.* (1991) also demonstrated that the sequence of chloroplast DNA intergenic spacers can be used for phylogenetic studies of closely related species. The removal of chloroplast data from the analysis did not alter the dendrogram obtained in the present study, thereby showing that the clustering obtained was really based on the presence of other *Coffea* species*.* This is also the case for Brazilian coffee variety clustering with interspecific genotypes. The Tupi variety is a hybrid between *C. arabica* and Híbrido do Timor. Icatu Vermelho comes from a cross between *C. canephora* and Bourbon Vermelho. Even though *C. canephora* is present in the genealogies of Oeiras, Catucaí Amarelo, Catucaí Vermelho and Obatã, these genotypes were grouped separate from interspecific hybrids. This might be due to differences in the background of the material used, or to the size of *C. canephora* introgressions in the varieties*.* The clustering of Brazilian varieties is in accordance with genealogical data. The varieties Acaiá Cerrado and Mundo Novo showed 100% similarity, which can be explained by Acaiá Cerrado being a selection inside Mundo Novo. In spite of the high genetic similarity among varieties, they are phenotypically different. In Acaiá Cerrado, tree-tops are cylindrical and diameter reduced when compared to Mundo Novo. Maluf *et al.* (2005) also found these two varieties to be identical. Genetic similarity among Ouro Verde, Rubi and Topázio is most probably due to the latter two having Catuaí as a parent, whereas Ouro Verde is a selection from Catuaí Amarelo. In Ouro Verde and Rubi, fruits are red and in Topázio yellow, whereas the young leaves of Ouro Verde are green and those of Rubi tanned.

The impossibility of separating color mutants, such as Catucaí Amarelo and Vermelho and Catuaí Amarelo and Vermelho, is to be expected, as mutants are usually the result of very few mutations that are difficult to spot with molecular markers (Weising *et al.*, 1995; Vosman and Arens, 1997). No polymorphism caused by mutation was observed with microsatellites in peaches (Testolin *et al.*, 2000), *Pelagonium* (Becher *et al.*, 2000) and roses (Esselink *et al.*, 2003; Vosman *et al.*, 2004). When a mutation occurs in any of the genes involved in the synthesis of color components, a color-mutant might be generated. In coffee, one gene involved in fruit-color formation is known, and two alleles (Xc and xc) have been identified (Mendes and Guimarães, 1998). Among the commercial *arabica* varieties, there are other morphological differences, such as plant height, leaf shape and size, leaf-color, branch-angle and stature. However, the differences among varieties at the DNA level are limited, probably due to several commercial arabica varieties originating either from single mutations or few ancestors.

According to the pairwise similarity matrix, genetic similarity was at least 0.860 in Obatã, Caturra Amarelo and Vermelho, Oeiras, Catuaí Amarelo and Vermelho and Bourbon Vermelho. Many of these varieties are known mutants or were obtained from selections or crosses between these varieties, as is shown in Table 1. Similar results have been recorded by many authors. Steiger *et al.* (2002) and Maluf *et al.* (2005) also observed high genetic similarity between Caturra and Catuaí. The Obatã variety is a selection from Sarchimor, itself originating from crossing Villa Sarchi with Híbrido do Timor. High genetic similarity was also observed between the varieties Villa Sarchi and Caturra (Anthony *et al.*, 2002). Thus Obatã was clustered with the above mentioned varieties.

The distance between Bourbon Vermelho and Amarelo is probably due to the latter being a natural cross between Bourbon Vermelho and the variety Amarelo de Botucatu (Mendes and Guimarães, 1998; Maluf *et al.*, 2005). Anthony *et al.* (2002) noted that the M-24 primer was useful for discriminating Bourbon from other varieties. In the present work the same primer amplified an allele that also facilitated the separation of Bourbon Amarelo from all other Brazilian varieties.

Probably through being derived from Sarchimor with *C.canephora* ancestry, IAPAR 59 remained clustered close to interspecific hybrids. Likewise, the varieties Icatu Amarelo, Icatu Vermelho and Tupi, also with *C.canephora* as a common ancestor, were clustered among interspecific material.

Irrespective of the high genetic similarity, a certain level of polymorphism is still to be found among *C. arabica* varieties, whereby hybrids with better performances have been obtained in Brazilian breeding programs. Heterosis reached 25% in hybrids between the varieties IAPAR 59 and Mundo Novo (Diniz *et al.*, 2005). This could be the result of the complementary action of simply a few genes.

The present fingerprint data generated for Brazilian varieties could be used to construct a DNA reference database for the molecular identification of varieties, as previously suggested (Bredemeijer *et al.*, 2002; Aggarwal *et al.*, 2004; Hendre *et al.*, 2008).

## Supplementary Material

The following online material is available for this article

Table S1 Sequences of the developed primers.

Table S2 Allelic profiles of the 19 coffee varieties and 6 interspecific hybrids obtained with polymorphic markers.

This material is made available as part of the online article from http://www.scielo.br.gmb.

## Figures and Tables

**Table 1:** Genealogy of the studied coffee varieties.

Number	Origin	Name	Background
1	Brazil	Acaiá Cerrado MG1474	Selection from Mundo Novo
2	Brazil	Mundo Novo IAC 376-4	Sumatra X Bourbon Vermelho
3	Brazil	Obatã IAC1669-20	Selection from Sarchimor^1^
4	Brazil	Oeiras MG 6851	Selection from Caturra Vermelho X Hibrido do Timor
5	Brazil	Ouro Verde IAC H5010-5	Selection from Catuaí Amarelo and Mundo Novo
6	Brazil	Rubi MG1192	BC Catuaí Vermelho X Mundo Novo
7	Brazil	Topázio MG1190	BC Catuaí Amarelo X Mundo Novo
8	Brazil	Bourbon Amarelo IAC J22	Típica
9	Brazil	Bourbon Vermelho IAC 662	Típica
10	Brazil	Catuaí Amarelo IAC 62	Selection Mundo Novo X Caturra Amarelo
11	Brazil	Catuaí Vermelho IAC 99	Mundo Novo X Caturra Amarelo
12	Brazil	Catucaí Amarelo 2015/ cova 479	Icatu Amarelo X Catuaí Vermelho
13	Brazil	Catucaí Vermelho 2015/cova 476	Icatu Vermelho X Catuaí Amarelo
14	Brazil	Caturra Amarelo IAC 476	Mutant of Bourbon Vermelho
15	Brazil	Caturra Vermelho IAC 477	Mutant of Bourbon Vermelho
16	Brazil	IAPAR 59	Selection from Sarchimor^a^
17	Brazil	Tupi IAC 1669-33	Selection from Sarchimor^a^
18	Brazil	Icatu Amarelo IAC 2944	Bourbon Amarelo X Icatu Vermelho
19	Brazil	Icatu Vermelho IAC 2945	Bourbon Vermelho X *C. canephora*
20	Portugal	CIFC H147/1	*C. arabica* X *C. liberica*
21	Portugal	CIFC 34/13 (S353-4/5)	*C. arabica* X *C. liberica*
22	Portugal	CIFC 832/1	Híbrido do Timor (*C. arabica* X *C. canephora*)
23	Portugal	CIFC 1343/269	Híbrido do Timor clone (*C. arabica* X *C. canephora*)
24	Portugal	CIFC 110/5	*C. arabica* X *C. Arabica*
25	Portugal	CIFC H539/8	*C. arabica* X *C. Canephora*

^1^Sarchimor = Villa Sarchi X Híbrido do Timor (= *C. arabica* X *C. canephora*).

**Table 2:** Results from microsatellite cloning and sequencing of two enriched libraries (EL) of *C. arabica*. Two elution conditions were used: 1) low-stringency (0.5xSSC) and 2) high-stringency (0.2xSSC). Positive clone indicates the number of clones hybridizing to a labeled oligo probe mixture. SSR indicates the number of clones containing a microsatellite. Designed primers indicate the number of clones on the basis of which primers could be designed for amplification of the microsatellite.

EL	Screened clones	Positive clones	Sequenced clones	SSR	Primers designed	Polymorphic markers
1	3572	135	110	45	18	2
2	3840	397	192	89	35	5

**Table 3:** Number of alleles per locus, number of effective alleles (ne) and number of allelic phenotypes from the 22 polymorphic microsatellite markers.

SSR marker	N. of alleles per locus	N. of effective alleles (ne)	N. of allelic phenotype
M20^1^	3	1.3	4
M24	4	1.9	5
CarM065^2^	1	1	1
CarM070	1	1	1
CarM069	2	1.1	2
CarM068	1	1	1
CarM086^3^	1	1	1
CarM092	3	1.9	4
CarM096	3	1.8	2
CarM101	7	3.9	4
CarM105	2	1.4	3
CarM001^4^	1	1	1
CarM002	1	1	1
CarM048^5^	5	1.8	6
CarM049	2	1.2	3
CarM050	2	1.5	2
CarM051	5	3.4	6
CarM052	7	1.8	7
Ccmp3^6^	1	1	1
Ccmp6	2	1.2	2
Ccmp10	1	1	1
NTCP8	1	1	1
Total number of alleles		55	
Average alleles/locus		2.5	

^1^Primer sequences published by Combes *et al.* (2000), ^2^Primers developed from clone sequences published by Rovelli *et al.* (2000) in the NCBI database; ^3^Primers developed from clone sequences published in the NCBI database; ^4^Primers obtained in the first genomic library; ^5^Primers obtained in the second genomic library; ^6^Chloroplast markers.
