# Supplementary material for: Development of microsatellite markers for identifying Brazilian Coffea arabica varieties
Source: Genet Mol Biol. 2010 Sep 1;33(3):507–14. doi: 10.1590/S1415-47572010005000055 (PMC3036114; doi:10.1590/S1415-47572010005000055)
Supplement: Table S1 — Sequences of the developed primers. [file gmb-33-3-515-suppl1.pdf]

Supplemental Table 1. Identification of the ERiCC accessions, their origin and common name.

	CNPAF Germplasm Bank Identification Number	Cultivation System*	Origin**	Common Name
1	CNA0000082	L	Suriname	AWINI
2	CNA0000122	L	India	ARC-10666
3	CNA0000586	L	Sri Lanka	BG 090-2
4	CNA0000692	L	India	BKN 6820-6-3-2
5	CNA0000754	L	Peru	CHANCAY
6	CNA0000798	L	Colombia	CICA 4
7	CNA0000923	L	Mexico	C 79-272-4-1-2-3-10
8	CNA0000950	L	India	IET 0355
9	CNA0000952	L	Pakistan	CR 36-148
10	CNA0001419	L	Colombia	COLOMBIA 1
11	CNA0001420	L	Colombia	CARREON
12	CNA0001423	L	Colombia	TAPURIPA-161
13	CNA0001467	L	India	IET 2881
14	CNA0002222	L	Lao	KH.YOUANE (V.T.A 13)
15	CNA0002246	L	Lao	KH.NGANH TAMAY (SVA 33)
16	CNA0002253	L	Lao	KH.KHAO BAY (VT-A 25)
17	CNA0002258	L	Thailand	KU 56-3
18	CNA0002293	L	Thailand	KU 94-2
19	CNA0002416	L	Liberia	LAC 12
20	CNA0002437	L	Liberia	LAC 28
21	CNA0002480	L	Cameroon	M 40
22	CNA0002482	L	Cameroon	M 44
23	CNA0002529	L	Philippines	MEHR
24	CNA0002672	L	Thailand	NAHNG PAYA 132
25	CNA0002871	L	Portugal	RIZZOTO 159
26	CNA0003195	L	Egypt	GZ 944-5-2-2
27	CNA0003196	L	Egypt	GZ 809-4-1-2
28	CNA0003005	U	India	110281
29	CNA0003241	L	India	PAU 41-306-2-1-PR 405
30	CNA0003411	L	Colombia	CICA 8
31	CNA0003417	L	Japan	PI 294351
32	CNA0003446	L	Philippines	IR 34
33	CNA0003452	L	-	CHIANUNG SEN 25

	CNPAF Germplasm Bank Identification Number	Cultivation System*	Origin**	Common Name
34	CNA0003569	L	India	KAU 2110
35	CNA0003591	L	India	TNAU 2686-1
36	CNA0003602	L	India	UPR 79-23
37	CNA0003665	L	Taiwan	KAOHSIUNG SEN YU 104
38	CNA0003668	L	Philippines	MTU 7029
39	CNA0004308	L	USA	CAROLINA SP 407
40	CNA0004552	L	Colombia	CR 1113
41	CNA0004566	L	Colombia	METICA 1
42	CNA0004576	L	Colombia	ZENIT
43	CNA0004579	L	Colombia	CAMPONI
44	CNA0004625	L	Colombia	TETEP
45	CNA0004629	L	Philippines	IR 50
46	CNA0005014	L	China	WU 10 B
47	CNA0005015	L	China	YAR AI ZHAO B
48	CNA0005016	L	China	ZHENSHAN 97 A
49	CNA0005477	L	China	K ASKHAM 36/14
50	CNA0005478	L	China	SZU MAIO
51	CNA0005853	L	Russia	WIR 5621
52	CNA0006910	L	Indonesia	MANINJAU
53	CNA0006943	L	Philippines	IR 54 R
54	CNA0006955	L	Italy	KORAL
55	CNA0006961	L	Italy	VITRO
56	CNA0007408	L	Colombia	WC 0144
57	-	L	Suriname	ELONI
58	-	L	Suriname	CIWINI
59	-	L	Suriname	CESWONI
60	-	L	Philippines	IR8
61	-	L	Philippines	IR36
62	-	L	USA	LEBONNET
63	-	L	India	BASMATI 370
64	-	L	Japan	NOURIN MOCHI
65	-	L	Japan	MINAMI HATA MOCHI
66	-	L	Japan	TOMOE MOCHI
67	-	L	Japan	MOGAMI CHIKANARI
68	-	L	Colombia	ORYZICA LHANOS4
69	-	L	Colombia	ORYZICA 1
70	-	L	Colombia	CICA 7
71	-	L	Colombia	CICA 9
72	-	L	Philippines	HUAN-SEN-GO
73	-	L	-	5287
74	-	L	-	RAMTULASI
75	CNA0000482	U	USA	BLUEBONNET
76	CNA0001006	U	USA	DAWN
77	CNA0002524	U	France	MOROBEREKAN
78	CNA0003287	U	France	IREM 123-2-1
79	CNA0003288	U	France	IREM 293-B

	CNPAF Germplasm Bank Identification Number	Cultivation System*	Origin**	Common Name
80	CNA0003289	U	France	IREM 247
81	CNA0003362	U	France	IRAT 142
82	CNA0003375	U	France	IRAT 13
83	CNA0003395	U	France	IRAT 141
84	CNA0003397	U	France	IRAT 144
85	CNA0003403	U	Nigeria	TOX 490-3-108-D1-B-B
86	CNA0004193	U	France	IREM 656
87	CNA0004428	U	France	N.7384 [RPL X DANIELA]
88	CNA0004463	U	France	N.7441 [CA 435 X TAINUNG CHUEN 2]
89	CNA0004480	U	France	IRAT 124
90	CNA0004487	U	France	MAKOUTA
91	CNA0004543	U	Nigeria	TOX 1012-12-3-1
92	CNA0004617	U	Nigeria	TOX 1011-4-2
93	CNA0004640	U	Nigeria	TOX 1785-19-18
94	CNA0004697	U	France	N.2583
95	CNA0004752	U	France	IRAT 122
96	CNA0004759	U	Nigeria	TOX 514-16-101-1
97	CNA0004788	U	Nigeria	TOX 503-4-115-B-B
98	CNA0004796	U	Nigeria	TOX 516-28-10B-D2-B-B
99	CNA0005277	U	Nigeria	TOX 1858-114
100	CNA0005326	U	Nigeria	TOX 1780-8-5
101	CNA0005334	U	Nigeria	TOX 1871-29
102	CNA0005358	U	Nigeria	TOX 1858-101
103	CNA0005970	U	Nigeria	FAROX 299
104	CNA0005972	U	Nigeria	FAROX 301
105	CNA0005994	U	Nigeria	TOX 995-208-1-101
106	CNA0006034	U	Nigeria	ITA 150
107	CNA0006035	U	Nigeria	ITA 225
108	CNA0006572	U	France	IREM 195
109	CNA0006574	U	France	IRAT 112
110	CNA0006940	U	USA	LEMONT
111	CNA0006941	U	USA	NEW BONNET
112	CNA0008092	U	France	L 141
113	CNA0008093	U	France	L 285
114	CNA0008411	U	USA	BLUE BELLE
115	CNA0008412	U	USA	BLUEBONNET 50
116	CNA0008432	U	USA	LACASSINE
117	CNA0008545	U	Colombia	CT 11216-10-12-B-BRM-10
118	CNA0009102	U	Colombia	CT10006-7-2-M-5-1P-3
119	CNA0009113	U	Colombia	CT10037-9-4-M-1-1P-2-M
120	CNA0009115	U	Colombia	CT11632-3-3-M
121	CNA0009123	U	Colombia	CT11891-3-3-3-M
122	CNA0009124	U	Colombia	CT13364-7-1
123	CNA0009139	U	Colombia	CT13366-15-4
124	CNA0009154	U	Colombia	CT13370-2-M

	CNPAF Germplasm Bank Identification Number	Cultivation System*	Origin**	Common Name
125	CNA0009197	U	Colombia	CT13377-8-4
126	CNA0009199	U	Colombia	CT13381-1-3
127	CNA0009223	U	Colombia	CT13569-5-7
128	CNA0009227	U	Colombia	CT13570-3-2
129	CNA0009240	U	Colombia	CT13572-6-2
130	CNA0009280	U	Colombia	CT13573-11-2
131	CNA0009319	U	Colombia	CT13579-3-4
132	CNA0009364	U	Colombia	CT13581-5-2
133	CNA0009415	U	Colombia	CT13582-11-4
134	CNA0009561	U	Colombia	CT13584-12-9
135	CNA0009591	U	Colombia	CT13585-12-3
136		U	-	B6149F-MR-19
137	-	U	Philippines	B8503-TB-19-B-3
138	-	U	-	BR4742-B-19-23
139	-	U	India	CUTACK 4
140	-	U	Philippines	IR65907-188-1-B
141	-	U	France	IRAT 10
142	-	U	Philippines	KATAKTARA
143	-	U	Philippines	TB154E-TB-2
144	-	U	Philippines	TB47H-MR-11-51-3
145	-	U	Philippines	YN1905-UUL-62
146	-	U	Philippines	YN906-UUL 65
147	-	U	China	YUNLU N 1
148	-	U	China	YUNLU N 7
149	-	L	EMBRAPA-CNPAF/ Brazil	RS16PL12-35-1-B
150	-	L	EMBRAPA-CNPAF/ Brazil	RS16PL1-34-4-B
151	-	L	EMBRAPA-CNPAF/ Brazil	RS16PL5-12-6-B
152	-	L	EMBRAPA-CNPAF/ Brazil	RS16PL12-10-1-B
153	CNA0001416	L	EMBRAPA-CNPAF/ Brazil	IPSL 0574
154	CNA0001117	L	EEPG/ Brazil	EEPG-1-169
155	-	L	EMBRAPA-CPACT/ Brazil	BRS AGRISUL
156	-	L	EMBRAPA-CPACT/ Brazil	BRS BOJURU
157	-	L	EMBRAPA-CNPAF/ Brazil	DIAMANTE
158	-	L	EMBRAPA-CNPAF/ Brazil	BRS FORMOSO
159	-	L	EMBRAPA-CNPAF/ Brazil	MARAJO
160	-	L	EMBRAPA-CNPAF/ Brazil	BRS JABURU
161	-	L	EMBRAPA-CNPAF/ Brazil	BRS BIGUA
162	-	L	EMBRAPA-CNPAF/ Brazil	RIO GRANDE
163	-	L	EPAGRI/ Brazil	EPAGRI 107
164	-	L	EPAGRI/ Brazil	EPAGRI 108
165	-	L	EPAGRI/ Brazil	SCS 111
166	-	L	EPAGRI/ Brazil	SCS 112
167	CNA0001337	L	IPEACO/ Brazil	IPEACO-SL 1969
168	CNA0001339	L	IPEACO/ Brazil	IPEACO-SL 0769
169	CNA0001344	L	IPEACO/ Brazil	IPEACO-SL 1469

	CNPAF Germplasm Bank Identification Number	Cultivation System*	Origin**	Common Name
170	CNA0001407	L	IPEACO/ Brazil	IPSL 2070
171	CNA0001413	L	IPEACO/ Brazil	IPSL 0970
172	CNA0001414	L	IPEACO/ Brazil	IPSL 0570
173	CNA0001106	L	IRGA/ Brazil	EEA 405
174	CNA0001107	L	IRGA/ Brazil	EEA 401
175	CNA0001108	L	IRGA/ Brazil	EEA 404
176	CNA0001109	L	IRGA/ Brazil	EEA 406
177	-	L	IRGA/ Brazil	IRGA 409
178	-	L	IRGA/ Brazil	IRGA 413
179	-	L	IRGA/ Brazil	IRGA 416
180	-	L	IRGA/ Brazil	IRGA 417
181	-	L	IRGA/ Brazil	IRGA 418
182	-	L	IRGA/ Brazil	IRGA 419
183	-	L	IRGA/ Brazil	IRGA 420
184	CNA0006129	L	EMPASC/ Brazil	EMPASC 103
185	CNA0006130	L	EMPASC/ Brazil	EMPASC 104
186	CNA0001118	U	EEPG/ Brazil	EEPG-1-269-FURNAS
187	CNA0000963	U	IPEACO/ Brazil	AMARELAO X GUEDES
188	CNA0000969	U	IPEACO/ Brazil	HONDURAS X MATAO
189	CNA0000976	U	IPEACO/ Brazil	SATURNO X PRATAO PRECOCE
190	CNA0000994	U	IPEACO/ Brazil	ESAV X MATAO
191	CNA0001347	U	IPEACO/ Brazil	IPEACO-SL 2270
192	CNA0001350	U	IPEACO/ Brazil	IPEACO-SL 1970
193	CNA0004098	U	EMBRAPA-CNPAF/ Brazil	XINGU
194	CNA0004120	U	EMBRAPA-CNPAF/ Brazil	RIO PARAGUAY
195	CNA0004121	U	EMBRAPA-CNPAF/ Brazil	GUARANI
196	CNA0004141	U	EMBRAPA-CNPAF/ Brazil	TRIUNFO
197	CNA0004172	U	EMBRAPA-CNPAF/ Brazil	GUAPORE
198	CNA0004206	U	EMBRAPA-CNPAF/ Brazil	ARAGUAIA
199	CNA0004748	U	EMBRAPA-CNPAF/ Brazil	CUIABANA
200	CNA0005180	U	EMBRAPA-CNPAF/ Brazil	TANGARA
201	CNA0006187	U	EMBRAPA-CNPAF/ Brazil	CAIAPO
202	CNA0006701	U	EMBRAPA-CNPAF/ Brazil	CARAJAS
203	CNA0007024	U	EMBRAPA-CNPAF/ Brazil	CNAX 1503-12-9-4-B
204	CNA0007119	U	EMBRAPA-CNPAF/ Brazil	AIMORE
205	CNA0007706	U	EMBRAPA-CNPAF/ Brazil	CONFIANCA
206	CNA0008070	U	EMBRAPA-CNPAF/ Brazil	PRIMAVERA
207	CNA0008711	U	EMBRAPA-CNPAF/ Brazil	SOBERANA
208	CNA0005673	U	IAC/ Brazil	IAC 81-176
209	CNA0005901	U	IAC/ Brazil	URUCUI
210	CNA0006170	U	IAC/ Brazil	LS 85-125
211	CNA0006174	U	IAC/ Brazil	LS 85-158
212	CNA0007799	U	IAC/ Brazil	IAC 1191
213	CNA0006406	U	IAC/ Brazil	LS 86-68
214	CNA0004168	U	IAPAR/ Brazil	L 80-68

	CNPAF Germplasm Bank Identification Number	Cultivation System*	Origin**	Common Name
215	CNA0005672	U	IAPAR/ Brazil	L 82-192
216	CNA0006666	U	IAPAR/ Brazil	A12-286-1-1
217	CNA0006672	U	IAPAR/ Brazil	A8-204-1-1
218	CNA0008309	U	IAPAR/ Brazil	L 92-342
219	CNA0006413	U	IAPAR/ Brazil	L 85-20
220	CNA0006422	U	IAPAR/ Brazil	IAPAR L 99-98
221	CNA0005650	U	IPEACO/ Brazil	IPEACO 11-P
222	CNA0005660	U	IPEACO/ Brazil	IPEACO 77-P
223	CNA0003281	U	Brazil	IRAT 177
224	CNA0005342	U	Brazil	RIO VERDE
225	CNA0002123	U	EEPG/ Brazil	JAPONES X PRAIANA
226	-	U	IAC/ Brazil	IAC 201
227	-	U	IAC/ Brazil	IAC 202
228	-	U	IAC/ Brazil	IAC 165
229	-	U	IAPAR/ Brazil	IAPAR 9
230	-	U	IAPAR/ Brazil	IAPAR 62
231	-	U	IAPAR/ Brazil	IAPAR 63
232	CNA0003490	U	Brazil	MEARIN
233	CNA0004078	U	Brazil	-
234	CNA0004243	U	Brazil	-
235	CNA0005975	U	Brazil	-
236	CNA0006030	U	EMBRAPA-CNPAF/ Brazil	-
237	CNA0007425	U	EMBRAPA-CNPAF/ Brazil	CANASTRA
238	CNA0007937	U	EMBRAPA-CNPAF/ Brazil	PROGRESSO
239	CNA0008172	U	EMBRAPA-CNPAF/ Brazil	BONANCA
240	CNA0008305	U	EMBRAPA-CNPAF/ Brazil	CARISMA
241	CNA0008533	U	EMBRAPA-CNPAF/ Brazil	MARAVILHA
242	CNA0008540	U	EMBRAPA-CNPAF/ Brazil	TALENTO

* L = lowland accessions ; U = upland accessions. For comparison with other studies, the lowland accessions used in this study were considered as indica and the upland accessions, as japonica [Khush GS (1997) Origin, dispersal, cultivation and variation of rice. Plant Molecular Biology 35:25-34].

** EMBRAPA – CNPAF = Empresa Brasileira de Pesquisa Agropecuária – Centro Nacional de Pesquisa (Brazil); IAC = Instituto Agronômico de Campinas (Brazil); IPEACO = Instituto de Pesquisa Agropecuária do Centro Oeste (Brazil); IAPAR = Instituto Agronômico do Paraná (Brazil); EMPASC = Empresa de Pesquisa Agropecuária de Santa Catarina (Brazil); EEPG = Estação Experimental de Ponta Grossa (Brazil); IRGA = Instituto Rio Grandense do Arroz (Brazil); EPAGRI = Empresa de Pesquisa Agropecuária e Extensão Rural de Santa Catarina (Brazil)
