# Supplementary material for: Development of microsatellite markers for identifying Brazilian Coffea arabica varieties
Source: Genet Mol Biol. 2010 Sep 1;33(3):507–14. doi: 10.1590/S1415-47572010005000055 (PMC3036114; doi:10.1590/S1415-47572010005000055)
Supplement: Table S2 — Allelic profiles of the 19 coffee varieties and 6 interspecific hybrids obtained with polymorphic markers. [file gmb-33-3-515-suppl2.pdf]

Supplemental Table 2. SSR Markers used for the evaluation of the ERiCC accessions (Data on “Marker concentration for multiplexed PCR”, “Missing data”, “Number of alleles” and “PIC” were obtained in this study).

Panel	SSR Marker	Marker concentration for multiplexed PCR (nM)	Missing data (%)	Fluorochrome	Allele size range (bp)	Number of alleles	PIC	Motif	Chromosome	References**
1	RM229	129	5.9	HEX	106–131	11	0.83	$(TC)_{11}(CT)_5C_3(CT)_5$	11	Chen et al. (1997)
	RM207	129	3.1	FAM	84–158	23	0.86	$(CT)_{25}$	2	Chen et al. (1997)
2	RM222*	129	10	FAM	199–215	12	0.72	$(CT)_{18}$	10	Chen et al. (1997)
	RM1	71.67	0.8	FAM	76-119	23	0.75	$(AG)_{26}$	1	Panaud et al. (1996)
	RM248	150	4.1	HEX	80–104	11	0.81	$(CT)_{25}$	7	Chen et al. (1997)
	RM38	71.67	5.7	HEX	246–278	18	0.86	$(GA)_{16}$	8	Chen et al. (1997)
3	RM5	71.67	1.8	FAM	109-133	9	0.73	$(GA)_{14}$	1	Panaud et al. (1996)
	RM253	71.67	9.8	HEX	89-119	11	0.75	$(GA)_{25}$	6	Chen et al. (1997)
4	RM11	71.67	3.6	HEX	123–143	11	0.78	$(GA)_{17}$	7	Panaud et al. (1996)
	RM13*	129	4.6	FAM	129–164	15	0.72	$(GA)_6$-$GA)_{16}$	5	Panaud et al. (1996)
5	RM252	129	11	FAM	193–277	23	0.62	$(CT)_{19}$	4	Chen et al. (1997)
	RM277	129	1.8	FAM	110–126	8	0.58	$(GA)_{11}$	12	Temnykh et al. (2000)
	RM204	129	6.9	HEX	106–194	32	0.87	$(CT)_{44}$	6	Chen et al. (1997)
6	RM231	129	10.5	FAM	170–196	10	0.78	$(CT)_{16}$	3	Chen et al. (1997)
	RM304	129	10	HEX	82–198	21	0.84	$(GT)_2(AT)_{10}(GT)_{33}$	10	Temnykh et al. (2000)

Panel	SSR Marker	Marker concentration for multiplexed PCR (nM)	Missing data (%)	Fluorochrome	Allele size range (bp)	Number of alleles	PIC	Motif	Chromosome	References**
7	OG17	71.67	2.3	FAM	114-180	24	0.77	$(CT)_{24}$	2	Brondani et al. (2001)
	OG44*	71.67	2.8	HEX	152-182	15	0.80	$(CT)_{4}$-23bp-$(CT)_{22}$-$(GT)_{4}$-$(GC)_{6}$	3	Brondani et al. (2001)
8	OG106*	129	10	FAM	200-250	22	0.90	$(CT)_{27}$	9	Brondani et al. (2001)
	4879	129	9.5	HEX	108-150	10	0.80	$(AAT)_{8}$	4	Rangel (2005)
9	4653*	129	3.9	FAM	80-170	19	0.85	$(AAG)_{25}$	12	Rangel (2005)
	RM224	129	4.1	HEX	124-158	10	0.81	$(AAG)_{8}$-$(AG)_{13}$	11	Chen et al. (1997)
10	RM223*	129	3.6	FAM	139-163	15	0.64	$(CT)_{25}$	8	Chen et al. (1997)
	RM257	71.67	4.1	HEX	130-192	22	0.90	$(CT)_{24}$	9	Chen et al. (1997)
11	OG61*	129	2.3	FAM	96-152	28	0.88	$(CT)_{18}$	5	Brondani et al. (2001)
	RM234	129	0.8	HEX	126-156	13	0.78	$(CT)_{25}$	7	Chen et al. (1997)
12	RM103	198	3.0	FAM	300-350	4	0.64	$(GAA)_{5}$	6	Temnykh et al. (2000)
	RM334	258	3.3	FAM	120-210	14	0.86	$(CTT)_{20}$	5	Temnykh et al. (2000)
	RM309	198	1.9	HEX	150-180	14	0.79	$(GT)_{13}$	12	Temnykh et al. (2000)
13	RM286	138	2.1	FAM	80-140	14	0.84	$(GA)_{16}$	11	Temnykh et al. (2000)
	RM276	180	3.9	HEX	75-160	17	0.84	$(AG)_{8}$-A_{3}-$(GA)_{33}$	6	Temnykh et al. (2000)

Panel	SSR Marker	Marker concentration for multiplexed PCR (nM)	Missing data (%)	Fluorochrome	Allele size range (bp)	Number of alleles	PIC	Motif	Chromosome	References**
14	RM125	129	0.3	FAM	100-150	13	0.58	$(GCT)_{8}$	7	Temnykh et al. (2000)
	RM171	71.67	7.5	FAM	310-344	8	0.59	$(GATG)_{5}$	10	Akagi et al. (1996)
	RM484	71.67	7.5	HEX	290-300	3	0.53	$(AT)_{9}$	10	Temnykh et al. (2001)
	RM341	71.67	1.0	HEX	110-180	19	0.76	$(CTT)_{20}$	2	Temnykh et al. (2000)
15	RM169*	258	0.3	FAM	150-200	14	0.75	$(GA)_{12}$	5	Temnykh et al. (2000)
	RM216	129	0.5	HEX	120-155	10	0.81	$(CT)_{18}$	10	Chen et al. (1997)
16	RM152	258	0.3	FAM	150-180	10	0.58	$(GGC)_{10}$	8	Akagi et al. (1996)
	RM267	129	0.3	HEX	125-175	11	0.49	$(GA)_{21}$	5	Temnykh et al. (2000)
17	RM264	180	3.3	FAM	125-195	22	0.83	$(GA)_{27}$	8	Temnykh et al. (2000)
	RM263	138	0.8	HEX	164-220	13	0.80	$(CT)_{34}$	2	Chen et al. (1997)
18	RM335	180	3.3	FAM	100-170	26	0.90	$(CTT)_{25}$	4	Temnykh et al. (2000)
	RM287	138	8.0	HEX	90-140	12	0.75	$(GA)_{21}$	11	Temnykh et al. (2000)
19	RM151	258	2.3	FAM	197-220	7	0.74	$(TA)_{23}$	1	Akagi et al. (1996)
	RM271	138	4.3	FAM	75-125	6	0.80	$(GA)_{15}$	10	Temnykh et al. (2000)
	RM336*	129	3.3	HEX	100-200	6	0.79	$(CTT)_{18}$	7	Temnykh et al. (2000)

Panel	SSR Marker	Marker concentration for multiplexed PCR (nM)	Missing Data (%)	Fluorochrome	Allele size range (bp)	Number of alleles	PIC	Motif	Chromosome	References**
20	RM51	270	14.0	FAM	120-150	9	0.77	$(GA)_{13}$	7	Chen et al. (1997)
	RM332	129	12.0	HEX	140-190	6	0.62	$(CTT)_5$-$(CTT)_{14}$	11	Temnykh et al. (2000)
21	RM310	116.67	3.3	HEX	80-130	9	0.84	$(GT)_{19}$	8	Temnykh et al. (2000)
	RM9	100	4.5	FAM	120-200	14	0.80	$(GA)_{15}GT(GA)_2$	1	Panaud et al. (1996)
	RM22*	132	1.3	HEX	150-230	7	0.55	$(GA)_{22}$	3	Panaud et al. (1996)
22	RM55	150	3.2	HEX	200-245	18	0.89	$(GA)_{17}$	3	Chen et al. (1997)
	RM266	210	8.8	FAM	100-150	13	0.79	$(GA)_{19}$	2	Temnykh et al. (2000)
	RM278	258	1.3	HEX	120-160	17	0.89	$(GA)_{17}$	9	Temnykh et al. (2000)
23	RM85	180	2.3	FAM	80-120	8	0.79	$(TGG)_5(TCT)_{12}$	3	Temnykh et al. (2000)
	RM117	258	4.2	FAM	190-220	12	0.71	$(AG)_7$	12	Temnykh et al. (2000)
	RM302	180	1.3	HEX	70-210	8	0.82	$(GT)_{30}(AT)_8$	1	Temnykh et al. (2000)
24	RM206	258	7.8	FAM	115-210	12	0.80	$(CT)_{21}$	11	Chen et al. (1997)
	RM159	258	3.1	HEX	220-280	9	0.75	$(GA)_{19}$	5	Temnykh et al. (2000)
25	OG99	71.67	2.3	FAM	110-160	7	0.19	$(CT)_4TT(CT)_4CC(CT)_3$ – 16pb – $(CT)_8$	3	Brondani et al. (2001)
	OG60	71.67	0.5	HEX	100-160	14	0.74	$(CT)_{21}(TCC)_2$	4	Brondani et al. (2001)

Panel	SSR Marker	Marker concentration for multiplexed PCR (nM)	Missing Data (%)	Fluorochrome	Allele size range (bp)	Number of alleles	PIC	Motif	Chromosome	References**
26	RM261	138	4.5	FAM	100-130	15	0.59	$C_9(CT)_8$	4	Chen et al. (1997)
	RM3	210	3.2	HEX	115-160	4	0.70	$(GA)_2GG(GA)_{25}$	6	Panaud et al. (1996)
27	RM220	150	2.4	FAM	90-135	9	0.69	$(CT)_{17}$	1	Chen et al. (1997)
	RM280	258	4.1	HEX	140-190	6	0.88	$(GA)_{16}$	4	Temnykh et al. (2000)
	RM7	210	3.0	FAM	160-195	15	0.85	$(GA)_{19}$	3	Panaud et al. (1996)
28	RM144*	258	9.5	FAM	200-250	15	0.75	$(ATT)_{11}$	11	Temnykh et al. (2000)
	RM243	129	4.2	FAM	85-130	10	0.75	$(CT)_{18}$	1	Chen et al. (1997)
	RM317	258	5.0	HEX	120-180	9	0.76	$(GC)_4(GT)_{18}$	4	Temnykh et al. (2000)
29	RM190*	150	0.5	FAM	100-130	11	0.75	$(CT)_{11}$	6	Akagi et al. (1996)
	RM205	210	0.5	HEX	115-170	14	0.78	$(CT)_{25}$	9	Chen et al. (1997)
	RM70	258	11.5	FAM	150-215	11	0.80	$(ATT)_{33}$	7	Chen et al. (1997)
30	RM14	270	10.0	HEX	160-120	9	0.81	$(GA)_{18}$	1	Panaud et al. (1996)
	5371*	258	8.4	FAM	150-210	5	0.83	$(GAA)_{14}$	12	Rangel (2005)
	RM178	258	4.3	HEX	110-140	8	0.69	$(GA)_5(AG)_8$	5	Temnykh et al. (2000)
31	RM210	129	1.3	FAM	115-175	15	0.87	$(CT)_{23}$	8	Chen et al. (1997)
	RM240	129	0.3	HEX	120-160	5	0.47	$(CT)_{21}$	2	Chen et al. (1997)

Panel	SSR Marker	Marker concentration for multiplexed PCR (nM)	Missing Data (%)	Fluorochrome	Allele size range (bp)	Number of alleles	PIC	Motif	Chromosome	References**
32	RM161	198	4.4	HEX	150-200	8	0.59	$(AG)_{20}$	5	Temnykh et al. (2000)
	RM135	198	1.3	FAM	100-140	5	0.55	$(CGG)_{10}$	3	Akagi et al. (1996)
	OG10*	258	0.0	HEX	40-130	21	0.81	$(CT)_{29}$	9	Brondani et al. (2001)
33	RM30	270	4.9	HEX	70-120	15	0.64	$(AG)_9A(GA)_{12}$	6	Panaud et al. (1996)
	RM154	258	5.3	HEX	140-210	14	0.91	$(GA)_{21}$	2	Akagi et al. (1996)
	RM119	258	7.0	FAM	150-185	6	0.75	$(GTC)_6$	4	Temnykh et al. (2000)
34	RM311	270	5.0	HEX	160-200	8	0.74	$(GT)_3(GTAT)_8(GT)_5$	10	Temnykh et al. (2000)
	RM53*	258	10.0	FAM	168-208	7	0.76	$(GA)_{14}$	2	Chen et al. (1997)
35	RM12	129	2.1	FAM	150-190	7	0.41	$(GA)_{21}$	12	Panaud et al. (1996)
	RM19	258	11.0	HEX	200-270	11	0.72	$(ATC)_{10}$	12	Panaud et al. (1996)
**Average**	-	-		-	-	12.4	0.75	-	-	-

* Markers with presence of PCR artifacts (especially stutters)

**References

Akagi H, Yokozeki Y, Inagaki A, Fujimura T (1996) Microsatellite DNA markers for rice chromosomes. Theor Appl Genet 93: 1071-1077.

Brondani C, Brondani RPV, Rangel PHN, Ferreira ME (2001) Development and mapping of Oryza glumaepatula derived microsatellite markers in the interspecific cross O. glumaepatula x O. sativa. Hereditas 134: 59-71.

Chen X, Temnykh S, Xu Y, Cho YG, McCouch SR (1997) Development of a microsatellite framework map providing genome-wide coverage in rice (*Oryza sativa* L). Theor Appl Genet 95: 553-567

Panaud O, Chen X, McCouch SR (1996) Development of microsatellite markers and characterization of simple sequence length polymorphism (SSLP) in rice (*Oryza sativa* L.) Mol Gen Genet 252: 597-607

Rangel, PN. Construção de mapa de ligação e mapeamento de QTLs utilizando marcadores SSRs, ESTs e SNPs em cruzamento interespecífico *Oryza glumaepatula* X *Oryza sativa*. Thesis, Universidade Federal de Goiás, 111 pp.

Temnykh S, Park WD, Ayres NM, Cartinhour S, Hauck N, Lipovich L, Cho YG, Ishii T, McCouch SR (2000) Mapping and genome organization of microsatellite sequences in rice (*Oryza sativa* L.). Theor Appl Genet 100: 697-712
